# Chromium nanoparticles improve bone turnover regulation in rats fed a high-fat, low-fibre diet

**DOI:** 10.1371/journal.pone.0300292

**Published:** 2024-05-08

**Authors:** Ewelina Cholewińska, Przemysław Sołek, Jerzy Juśkiewicz, Bartosz Fotschki, Wojciech Dworzański, Katarzyna Ognik

**Affiliations:** 1 Faculty of Animal Sciences and Bioeconomy, Department of Biochemistry and Toxicology, University of Life Sciences in Lublin, Lublin, Poland; 2 Institute of Animal Reproduction and Food Research, Polish Academy of Sciences, Olsztyn, Poland; 3 Chair and Department of Human Anatomy, Medical University of Lublin, Lublin, Poland; Icahn School of Medicine at Mount Sinai Department of Pharmacological Sciences, UNITED STATES

## Abstract

The aim of the study was to investigate the effect of returning to a balanced diet combined with chromium picolinate (CrPic) or chromium nanoparticles (CrNPs) supplementation at a pharmacologically relevant dose of 0.3 mg/kg body weight on the expression level of selected genes and bone turnover markers in the blood and bones of rats fed an obese diet. The results of the study showed that chronic intake of a high-fat obesogenic diet negatively affects bone turnover by impairing processes of both synthesis and degradation of bones. The switch to a healthy diet proved insufficient to regulate bone metabolism disorders induced by an obesogenic diet, even when it was supplemented with chromium, irrespective of its form. Supplementation with CrPic with no change in diet stimulated bone metabolism only at the molecular level, towards increased osteoclastogenesis (bone resorption). In contrast, CrNPs added to the high-fat diet effectively regulated bone turnover by increasing both osteoblastogenesis and osteoclastogenesis, with these changes directed more towards bone formation. The results of the study suggest that unfavourable changes in bone metabolism induced by chronic intake of a high-fat diet can be mitigated by supplementation with CrNPs, whereas a change in eating habits fails to achieve a similar effect.

## Introduction

The structure andfunctioning of bone tissueare unquestionably determined by genetic factors, but environmentalfactors, such as ethnic origin, age, sex, height, body weight, and puberty, play an important role as well. The condition of the skeleton also seems to be clearly determined by the daily diet [1]. The literatureindicates that the increasing prevalence of the high-fat Western diet is not without effect on bone turnover ftcp1-3]. There are indications thatbone-formation processes may be stimulated by excessive adipose tissue depositedduring intake of a high-fat diet, through the increasedmechanical loadand endocrine activity involving the secretion of significant amounts of adipokines, such as leptin and adiponectin, which leads to peak bone mass and reduces the risk of osteoporosis [2,3]. In recent years, however, an increasing number of reports have suggested that excessive fat in the diet impairs bone mineralization and exacerbates the loss of bone mass, leading to a marked deterioration of the quality and mechanical properties of the skeleton [1]. The potential mechanismsof the negative effect of a high-fat diet on bone structure and health are assumed to be associated with its capacity to disturb the balance of the intestinal microbiota, impair the intestinal barrier, and induce inflammation and oxidative stress or accumulation of adipose tissue in the bone marrow [4]. The harmful impact of a high-fat diet onbone tissuemay also be due to the fact thatchronic fat intake induces deficiencies of important nutrients for boneremodeling such as Ca, P and vitamin D [5], and also leads to hormonalimbalance, manifested in part by reducedlevels of parathormone (PTH), which regulates the calcium–phosphate balance in the body [6].

The literature indicates that harmful imbalances between bone synthesis and resorption induced bychronicintake ofa high-fat diet can be mitigated by a change to healthier eating habits and appropriate medication [4]. In recent years, diet supplementation with Cr(III) compounds has increasingly been used to treat overweight and obesity. Studies have shown that by improving insulin signalling, this microelement improves carbohydrate and lipid metabolism, which translates to a reduction in body weight [7]. Moreover, theliterature shows that the addition of Cr(III) to the dietincreases Ca retention, indicating improvement of bone mineralization [8]. In addition, chromium beneficially modulates osteocalcin levels [9] and the activity of alkaline phosphatase (ALP) andtartrate-resistant acid phosphatase (TRAP), thereby preventing the development of osteoporosis [10].

Only a small portion (about 0.5–2%) of chromium occurring in the form of inorganic compounds is absorbed from the gastrointestinal tract, while most of it is excreted from the body. The use of organic forms of chromium, such aspicolinate, significantly increases its bioavailability, even up to 10–25% [11]. It is also believed that the use of Cr in the form of very small nanoparticles can increase its absorption from the gastrointestinal tract, therebyenhancing its effect on the body. However, this entails a certain risk, as excessive Cr can have toxic effects on the body,e.g. by increasing oxidation processes or damaging genetic material [7]. Therefore it may be assumed that if an excessive amount of Cr(III) is introduced to the body via the diet, it cannot improve bonefunctioning and structure, but only impair it [10]. The results of our previous studies in chickens and rats [12,13] have shown that Cr added to the diet can accumulate in the bones while reducing their content of P. In addition, Saeed et al. [14] report that Cr can displace other elements,including Ca, from the bone, becoming incorporated in their place, which negatively affects the properties of thebone tissue. Moreover, by increasing insulin activity in the bones, Cr can inhibit resorption processes by blocking the effects of parathormoneon osteoblasts [8].

In the present study, it was postulated that bone metabolism disorders induced by long-term intake of an obesogenic diet can be mitigated by switching to a balanced low-fatdiet and/or supplementing the diet witha pharmacologically relevant dose of chromium(III), eitherin the standard form of picolinate (CrPic) or as nanoparticles (CrNPs).The aim of the study was therefore to verify this hypothesis by determining the effect of a return to a balanced diet in combination with supplementation with CrPic or CrNPs in the amount of 0.3 mg/kg of body weight on the level of expression of selected genes and markers of bone turnoverin the blood and bones of rats fed an obesogenic diet.

## Material and methods

The study presented in this paper is part of a large research project aimed at determining the effects of a return to a balanced diet in combination with supplementation with CrPic or CrNPs on multiple aspects of the biological response in rats with obesity induced by a high-fat, low-fibre diet. Therefore both the design of the experiment and the procedures used have previously been published in works by Stępniowska et al. [13], Stępniowska et al. [15], Fotschki et al., [16] and Fotschki et al. [17].

### Characterization of chromium forms

Chromium nanoparticles (CrNPs) were purchased from Sky Spring Nanomaterials Inc. (Houston, TX, USA).They were 60–80 nm in size (nanopowder) and had purity of 999 g/kg, spherical morphology, a specific surface area of 6–8 m^2^/g, 0.15 g/cm^3^ bulk density, and 8.9 g/cm^3^ true density. Chromium picolinate (CrPic), with purity >980 g/kg, was purchased from Sigma-Aldrich Co. (Poznań, Poland).

### Animal protocol and dietary treatments

All animal care and experimental protocols were in compliance with current laws governing animal experimentation in the Republic of Poland and the European Convention for the Protection of Vertebrate Animals used for Experimental and other Scientific Purposes, Directive 2010/63/EU [18] for animal experiments, and were approved by the National Ethics Committee for Animal Experiments (Approval No. 73/2021).

The *in vivo* experiment was performed using 84 outbred male Wistar rats (Cmdb:Wi CMDB) fed a standard low-fat or high-fat/low-fibre (obesogenic) semi-purified rat diet without or with the dietary addition of chromium in one of two forms–picolinate (CrPic) and nanoparticles (CrNPs) (Table 1). Rats were housed randomly and individually in stainless steel cages under a stable temperature (22 ± 1°C), relative humidity 60 ± 5%, a 12 h light–dark cycle, and a ventilation rate of 15 air changes per hour. Throughout the study all animals were monitored daily for any abnormal rat behaviour or indicators of fear, distress, pain, or anxiety, so that humane endpoints could be identified. For 18 weeks (9-week initial and 9-week experimental period), the rats had free access to tap water and semi-purified diets, which were prepared and then stored at 4°C in hermetic containers until the end of the experiment. The diets were modifications of a casein diet for laboratory rodents recommended by the American Institute of Nutrition [19].

**Table 1:** The composition of the experimental diets (g/100 g).

	C	F	MP	FP	MN	FN
Casein	14.8	14.8	14.8	14.8	14.8	14.8
DL-Methionine	0.2	0.2	0.2	0.2	0.2	0.2
Cellulose	8	3	8	3	8	3
Choline chloride	0.2	0.2	0.2	0.2	0.2	0.2
Cholesterol	0.3	0.3	0.3	0.3	0.3	0.3
Vitamin mix^1^	1	1	1	1	1	1
Mineral mix^2^	3.5	3.5	3.5	3.5	3.5	3.5
Maize starch	64	52	64	52	64	52
Rapeseed oil	8	8	8 (with Cr-Pic)	8 (with Cr-Pic)	8 (with Cr-NP)	8 (with Cr-NP)
Lard	0	17	0	17	0	17

^1^AIN-93G-VM [19], g/kg mix: 3.0 nicotinicacid, 1.6 Ca pantothenate, 0.7 pyridoxine-HCl, 0.6 thiamin-HCl, 0.6 riboflavin, 0.2 folicacid, 0.02 biotin, 2.5 vitamin B-12 (cyanocobalamin, 0.1% in mannitol), 15.0 vitamin E (all-rac-α-tocopherylacetate, 500 IU/g), 0.8 vitamin A (all-trans-retinylpalmitate, 500,000 IU/g), 0.25 vitamin D-3 (cholecalciferol, 400,000 IU/g), 0.075 vitamin K-1 (phylloquinone), 974.655 powderedsucrose.

^2^Mineral mix, g/kg mix: 357 calciumcarbonateanhydrous CaCO_3_, 196 dipotassiumphosphate K_2_HPO_4_, 70.78 potassiumcitrate C_6_H_5_K_3_O_7_, 74 sodiumchloride NaCl, 46.6 potassiumsulfate K_2_SO_4_, 24 magnesiumoxideMgO, 18 microelementmixture, starch to 1 kg = 213.62. Microelementmixture, g/kg mix: 31 iron (III) citrate (16.7% Fe), 4.5 zinccarbonate ZnCO_3_ (56% Zn), 23.4 manganese (II) carbonate MnCO_3_ (44.4% Mn), coppercarbonate CuCO_3_ (55.5% Cu), 0.04 potassiumiodide KI, citricacid C_6_H_8_O_7_ to 100 g.

In our study, only male rats were included to our study to eliminate the influence of additional variables such as hormonal balance. The selected animal model was standardized and validated for the specific condition of diet-induced obesity. The model involved the administration of an obesogenic high-fat diet with low levels of fiber to induce obesity in a controlled and reproducible manner. The study schema consisted of two periods, initial and experimental, lasting 9 weeks each (Table 2). During the initial 9-week period, rats aged 6 weeks were randomly assigned to the control group (n = 12), fed a standard low-fat C diet,or the HF group (n = 72), fed an obesogenic diet. After the initial period, the rats from the control group C were fed the same standard low-fat C diet for the next 9 weeks of the experimental period. The HF rats were then randomly divided into 6 groups with n = 12 per group. Group M received a standard low-fat diet (the same diet as in the control group; this treatment imitates a change in the eating habits of an obese consumer without chromium supplementation, i.e. switching from a high energy density diet to a low-fat diet), while group F continued to be fed the obesogenic diet for the next 9 weeks (imitating the absence of changes in the eating habits of an obese consumer). Group MP was fed a standard low-fat diet supplemented with CrPic (imitating a switch from an obesogenic diet to a low-fat diet together with a common form of Cr); group MN was fed a standard low-fat diet supplemented with CrNPs (mirroring a switch from an obesogenic diet to a low-fat diet together with a novel form of Cr); group FP received an obesogenic diet supplemented with CrPic (imitating obese consumers who make no changes in basic dietary patterns but are aware of their unhealthy eating habits and willing to take a common Cr supplement); and group FN was fed an obesogenic diet supplemented with CrNPs (imitating obese consumers who make no changes in basic dietary patterns but areaware of their unhealthy eating habits and willing to take a novel Cr supplement). Groups C, MP, and MN were fed diets with 15.1, 21.5, and 63.4 kcal% from protein, fat, and carbohydrates, respectively. The corresponding values for diets F, FP, and FN were 11.6, 48.8, and 39.6 kcal%. Besides enhanced body weight of rats fed high-fat diet in the initial period, the obese state was confirmed by NMR analysis. The living rats were subjected to time-domain nuclear magnetic resonance using a minispec LF 90II analyser (Bruker, Karlsruhe, Germany) to determine the fat and lean tissue mass. The minispec transmits various radio frequency pulse sequences into soft tissues to reorient the nuclear magnetic spins of the hydrogen and then detects radio frequency signals generated by the hydrogen spins from these tissues. The contrast in relaxation times of the hydrogen spins found between adipose tissue and water-rich tissues is used to estimate fat and lean body mass. After the initial 9 wk period, the control rats were characterized by fat tissue (26.4%) and lean tissue (54.5%) contents, while in the rats fed high-fat diet those values were fat tissue (32.2%) and lean tissue (46.9%). The amount of chromium administered to each rat (groups MP, FP, MN, and FN) was 0.3 mg/kg body weight (BW), selected according to the EFSA NDA Panel (2014) [20]. The dosage should be considered the pharmacologically relevant dose of additional Cr in the diet. Taking advantages of our own experience with the formulation of diets containing nanoparticles and for the safety of the operator preparing the experimental diets with CrNPs, both Cr sources (to maintain comparable conditions) were added to the diet as an emulsion together with dietary rapeseed oil.

**Table 2:** Schema of the experimental groups (n = 12).

Experimental gruop	Initial 9 wks period		Experimental 9 wks period	
	Type of diet	Cr additional	Type of diet	Cr additional
**C (control)**	**C diet** (standard low-fat diet)	**without Cr supplementation**	**C diet** (standard low-fat diet)	**without Cr supplementation**
**M**	**F diet** (high-fat/low-fibre (obesogenic) diet)	**without Cr supplementation**	**C diet** (standard low-fat diet)	**without Cr supplementation**
**F**	**F diet** (high-fat/low-fibre (obesogenic) diet)	**without Cr supplementation**	**F diet** (high-fat/low-fibre (obesogenic) diet)	**without Cr supplementation**
**MP**	**F diet** (high-fat/low-fibre (obesogenic) diet)	**without Cr supplementation**	**C diet** (standard low-fat diet)	**CrPic** (chromium picolinate; 0.3 mg/kg BW)
**FP**	**F diet** (high-fat/low-fibre (obesogenic) diet)	**without Cr supplementation**	**F diet** (high-fat/low-fibre (obesogenic) diet)	**CrPic** (chromium picolinate; 0.3 mg/kg BW)
**MN**	**F diet** (high-fat/low-fibre (obesogenic) diet)	**without Cr supplementation**	**C diet** (standard low-fat diet)	**CrNPs** (chromium nanoparticles; 0.3 mg/kg BW)
**FN**	**F diet** (high-fat/low-fibre (obesogenic) diet)	**without Cr supplementation**	**F diet** (high-fat/low-fibre (obesogenic) diet)	**CrNPs** (chromium nanoparticles; 0.3 mg/kg BW)

All physiological measurements were performed separately for each animal (n = 12 for each group). At the end of the experiment, the rats were fasted for 12 h and anaesthetized *i*.*p*. with ketamine and xylazine (K, 100 mg/kg BW; X, 10 mg/kg BW) according to recommendations for anaesthesia and euthanasia of experimental animals. Next, following laparotomy, blood samples were taken from the caudal vena cava into heparinized tubes, and the rats were euthanized by cervical dislocation. Then the lefthindlegs were removed. Blood plasma was obtained by centrifugation (350×g, 10 min, 4°C) and kept frozen at −80°C until analysis. The left femurs were immediately removedfrom the legs, cleaned of tissues, and prepared for further analysis according to procedures described byNance et al. [21]. The prepared boneswere divided into two equal partsand then snap-frozen in liquid nitrogen and stored at −80°C prior to RNA extraction and preparation of homogenates.

### Laboratory analyses

#### Determination of bone marker levels in the plasma and bone homogenates by immunoenzymatic techniques

The analyses were begun by preparingbone homogenatesaccording to the protocol recommended by the manufacturer of the ELISA kits (Shanghai Qayee Biotechnology Co., Ltd., Shanghai, China)used to determine the levels of bone markers. Bone halves were placed in separate sealed aluminium foil packs and crushed with a hammer to obtain small fragments. Then 100–150 mg from each bone was weighed out and transferred to2 ml homogenization tubes containing steelhomogenizer beads (VWR International LLC, Radnor, Pennsylvania, USA) in the number recommended by the manufacturer.Then PBS withpH 7.4 was added to the tubes in an amount such that the ratio of bone mass toPBS volume was 1:9. All steps were performed on ice to preventdegradation of the organic components to be analysed. Samples prepared in this manner were homogenizedin VWR BEAD MILL MAX homogenizer (VWR International LLC, Radnor, Pennsylvania, USA) with integrated cooling, applying six20-secondcyclesat 5.5 m/s. The resulting homogenateswere transferred to 1.5 ml Eppendorf tubes, avoiding transferringthe homogenizer beads, and then centrifuged for 10 minat 3000xg, 4°C. Then the supernatant was removed and used for further analyses.

Next, the blood plasma and homogenates were analysed for expression of bone alkaline phosphatase (BALP), interferon-γ (IFN-γ), macrophage colony-stimulating factor (M-CSF), osteocalcin (OC), osteonectin (OCN), receptor activator of nuclear factor-κβ (RANK), receptor activator of nuclear factor-κβ ligand (RANKL), osteoprotegerin (OPG), prostaglandin E2 (PGE2), calcitonin (Ctn), parathyroid hormone (PTH), and the active form of vitamin D_3_ (vit. D). These parameters were determined using a commercial enzyme-linked immunosorbent assay (ELISA) kit, following the protocol provided by the manufacturer (Shanghai Qayee Biotechnology Co., Ltd., Shanghai, China). Absorbancewas measured at 450 nm with an ELISA reader (SunriseTM, Tecan Group Ltd., Männedorf, Switzerland). In addition, the RANKL:OPG ratio was calculated from the results.

#### RNA extraction and quantitative real-time PCR

As in the case of the bone homogenates, thepreviously prepared femur halveswere placed in sealed aluminium foil packs and crushed with a hammer to obtain small fragments. A portion of each crushed femur (about 100 mg) was transferred to a sterile 2 mL homogenization tubewith steel homogenizer beads (VWR International LLC, Radnor, Pennsylvania, USA), and1 mL Trizol Reagent (Thermo Fisher Scientific, Waltham, MA, USA) was added.All steps were performed on ice to prevent RNA degradation. The samples werehomogenizedin VWR BEAD MILL MAX homogenizer (VWR International LLC, Radnor, Pennsylvania, USA) with integrated cooling, applying five 20-second cycles at 5.5 m/s. Then RNA was extracted from the femurs according to the protocol provided by the manufacturer of Trizol Reagent. The isolated RNA yield was estimated spectrophotometrically (Nabi UV-VIS spectrophotometer, MicroDigital Co. Ltd., Gyeonggi, Republic of Korea), with integrity assessed electrophoretically by separation on 0.8% agarose gel. For complementary cDNA synthesis, 1 μg of RNA was reverse-transcribed using the NG dART RT kit (EURX Ltd., Gdańsk, Poland), according to the manufacturer’s instructions. Specific primers for evaluation of gene expression–Sp7 Transcription Factor (*SP7*), Runx2 Transcription Factor (*RUNX2*) and cathepsin K (*CTSK*)–were designed using Primer 3 software (Whitehead Institute, Cambridge, MA, USA) and synthesized by Genomed (Warsaw, Poland). The selection criteria for the analyzed genes and the corresponding primers were based on their relevance to bone metabolism and turnover, aiming to capture a comprehensive picture of the molecular changes in response to the experimental dietary conditions. *Sp7* (Osterix) was chosen for its role as a key transcription factor in osteoblast differentiation. *Runx2* (Core-binding factor subunit alpha-1) was elected due to its essential function in osteoblast differentiation and bone formation. *Cathepsin K* (CTSK) was included as it is a prominent marker for osteoclast activity and bone resorption. The overarching goal in selecting these genes for study was to capture key aspects of both osteoblast and osteoclast activity to provide a comprehensive understanding of the effects on bone turnover. The primers were designed to ensure specificity and efficiency in amplifying the target genes. The sequences of primers are shown in Table 3.

**Table 3:** Primer sequences for analysed genes.

Gene	Primer	Sequence (5′-3′)	Tm (°C)	Product size (nt)	Gen Bank access no.
*ACTB*	Forward	CCCGCGAGTACAACCTTCTTG	61,27	71	NM_031144.3
	Reverse	GTCATCCATGGCGAACTGGTG	61,61		
*GAPDH*	Forward	CCGCATCTTCTTGTGCAGTG	59.83	79	NM_017008.4
	Reverse	CGATACGGCCAAATCCGTTC	59.42		
*SP7*	Forward	CGGCCTGAGAGAGGAGAAGA	60.40	138	NM_001037632.1
	Reverse	GGACTGGAGCCATAGTGAGC	59.89		
*RUNX2*	Forward	GCGCATTCCTCATCCCAGTA	59.89	138	NM_001278483.2
	Reverse	GGTGGGGAGGATTGTGTCTG	60.04		
*CTSK*	Forward	AGAACTATGGCTGTGGAGGC	59.46	135	NM_031560.2
	Reverse	CCTTTGCCGTGGCGTTATAC	59.62		

Real-time PCR was performed on the Quantabio thermocycler (VWR International LLC, Radnor, Pennsylvania, USA) using SG qPCR Master Mix (2x) (EURX Ltd., Gdańsk, Poland) according to the following protocol: one cycle at 95°C for 10 min (initial denaturation), followed by a PCR including 38 cycles at 95°C for 25 s (denaturation), 59–61°C for 25 s (annealing), and 72°C for 40 s (elongation). A melting curve analysis was performed over 50–72°C at 0.3°C/s intervals. Negative controls without the cDNA template were provided. Real-time PCR was performed in duplicate. Normalized gene expression was calculated using the 2-ΔCt method. The glyceralde-hyde-3-phosphate dehydrogenase (*GAPDH*) and β-actin (*ACTB*) genes were selected as endogenous controls to normalize gene expression.

### Statistics

STATISTICA software, version 12.0 (StatSoft Corp., Krakow, Poland), was used to determine whether variables differed among treatment groups. Two-way ANOVA and Student’s *t*-test were used to analyze the results. Each experimental group was compared to the control C group with the aid of *t*-test (M vs. C; F vs. C; MP vs. C; FP vs. C; MN vs. C; FN vs. C). The results obtained from all experimental groups (M, F, MP, FP, MN, FN) were analyzed using two-way ANOVA. The two-way ANOVA was applied to assess the effects of the main factors: diet type (D; low-fat, high-fat/low fibre), additional Cr type (Cr; without, picolinate, nanoparticles), and the interaction between them (Cr×D). When ANOVA indicated significant interactioneffects, the means were evaluated using Duncan’s multiple range test. In that case, the differences between groups (M, F, MP, FP, MP, MN) were indicated in Tables with superscripts only in the case of a statistically significant interaction Cr×D (*P*≤ 0.05). The data were checked for normality prior to the statistical analysis. Differences at *P* ≤ 0.05 were considered significant.

## Results

### t-test

The t-test was applied to compare the experimental groupsM, F, MP, FP, MN and FN with the control group C at *P*< 0.05. When compared to the C rats, ahigher initial body weight of rats entering the experimental feeding period was observed, while the final body weight was higher in rats F, FP, MN and FN versus C [16]. Moreover, t-test showedlowerBALP levelin the plasma of rats from groups F, MN and FN than in the control group C.In addition, in comparison with group C, lower levels of IFN-γ, RANKL,PTHandvit. Dwere noted in the plasma of rats fromexperimental groups F, MP, FP, MN and FN. Lower plasma levels of Ctn and M-CSF were noted in rats from group MN. In comparison to group C, the plasma level of OC was lower in rats fromgroups M, F, MP, FP and MN, while thePGE2 level was lower in groups MN and FN, andthe RANK level was lower ingroups F, FP and MN.In addition, the RANKL:OPG ratio in the plasma of rats ingroups M and MP was increased, while in groupFN it was decreased relative to group C (Table 4). Lower BALP level was also noted in thebone tissueof rats fromgroups M and FN. In the bones of rats ingroups M, F, MP, FP, MN and FN, the IFN-γ and RANK levels were lower than in the control group, while the PTH level was higher. The Ctn level was also higher in thebonesof rats inexperimental groupsF, FP, MN and FN. The M-CSF level was lower in thebonesof rats ingroups MP and FN.OPG levels were lower in thebonesof rats fromexperimental groups F, MP, FP, MN and FN, while the RANKL level was higher. The bones of rats in groups F, MP, FP, MN and FN also had a higher RANKL:OPG value than in group C. Higher PGE2 content was noted in the bones of rats in group MN, and the vit. D level was lower in the bones of rats from group M (Table 5). Compared to the control group C, the bones of all experimental rats showed lower expression of the *SP7* gene. Relative to the control group, expression of the *CTSK* gene was reduced only in the bones of rats from groups M, F and MN, while that of the *RUNX2* gene was reduced in rats from groups M, MN and FN (Table 6).

**Table 4:** Plasma metabolism parameters of rats fed experimental diets* (n = 12).

	BALP	IFN- γ	OC	OCN	M-CSF	PGE2	RANK	RANKL	OPG	RANKL:OPG	Ctn	PTH	Vit D
	ng/mL	pg/mL	pg/mL	ng/mL	ng/mL	pg/mL	pg/mL	ng/mL	ng/mL		pg/mL	pg/mL	ng/mL
Control C	154	561	967	60.3	200	55.7	585	262	50.3	5.21	659	218	30.9
t-test/2-way ANOVA:													
M	138	519a	652^b#^	55.8	181	52.1	504^a^	226	38.9	5.81#a	661	200	29.2
F	120^#^	478^ab#^	653^b#^	53.7	178	44.5	436^b#^	205^#^	37.2	5.51ab	621	172^#^	25.8^#^
MP	137	465^abc#^	754^b#^	57.8	174	50.3	480^ab^	210^#^	33.9	6.20#a	648	171^#^	26.0^#^
FP	141	401^c#^	710^b#^	58.5	168	42.7	423^b#^	184^#^	33.8	5.44b	577	153^#^	23.3^#^
MN	110^#^	394^c#^	690^b#^	56.5	163^#^	36.1^#^	476^ab#^	172^#^	32.1	5.36b	559^#^	146^#^	21.6^#^
FN	123^#^	443^bc#^	965^a^	57.8	173	37.2^#^	587^a^	170^#^	39.6	4.29#c	590	155^#^	22.9^#^
*SEM*	*4*.*177*	*10*.*831*	*29*.*035*	*1*.*456*	*4*.*097*	*1*.*869*	*15*.*214*	*7*.*210*	*3*.*186*	*0*.*098*	*12*.*967*	*5*.*259*	*0*.*740*
Cr (additional)													
W (without)	129	499	653	54.7	180	48.3^a^	470	216^a^	38.1	5.66a	641	186^a^	27.5^a^
P (picolinate)	140	433	737	58.4	170	46.8^a^	454	197^ab^	33.7	5.82a	614	162^ab^	24.7^ab^
N (nano)	116	419	828	57.1	168	36.6^b^	531	171^b^	35.8	4.83b	574	150^b^	22.2^b^
*P value*	*0*.*095*	*0*.*003*	*0*.*041*	*0*.*672*	*0*.*551*	*0*.*033*	*0*.*100*	*0*.*035*	*0*.*874*	*0*.*012*	*0*.*156*	*0*.*015*	*0*.*019*
D (Diet type)													
M	129	460	702	56.8	173	46.4	488	203	34.9	5.79	624	172	25.6
F	128	441	776	56.7	173	41.5	482	186	36.9	5.08	596	160	24.0
*P value*	*0*.*932*	*0*.*335*	*0*.*182*	*0*.*962*	*0*.*971*	*0*.*207*	*0*.*836*	*0*.*245*	*0*.*773*	*0*.*056*	*0*.*330*	*0*.*211*	*0*.*273*
Interaction Cr×D													
*P value*	*0*.*348*	*0*.*051*	*0*.*039*	*0*.*911*	*0*.*745*	*0*.*555*	*0*.*031*	*0*.*761*	*0*.*851*	*0*.*324*	*0*.*307*	*0*.*295*	*0*.*362*

*The feeding period consisted of an initial 9-wk and an experimental 9-wk period. During the initial period the rats in group C were fed a diet with 8% rapeseed oil and 64% maize starch (diet C), while the remaining groups received a high-fat diet with 8% rapeseed oil, 17% lard, and 52% maize starch (diet F). Dietary treatments used in the experimental period: Group C, control fed diet C; M, fed diet C; F, fed diet F; MP, fed diet Csupplemented with chromium picolinate (CrPic; 0.3 mg/kg body weight (BW)); FP, fed diet F supplemented with CrPic (0.3 mg/kg BW); MN, fed diet C supplemented with chromium nanoparticles (CrNPs; 0.3 mg/kg BW); FN, fed diet F supplemented with CrNPs (0.3 mg/kg BW); W, treatment (n = 24) without Cr supplementation; P, treatment (n = 24) with CrPic supplementation; N, treatment (n = 24) with CrNPs supplementation; M, treatment (n = 36) with standard diet C; F, treatment (n = 36) with high-fat diet F. ^a,b^ Mean values within a column with different superscript letters are significantly different (*P*< 0.05); differences between groups (M, F, MP, FP, MP, MN) are indicated with superscripts only in the case of a statistically significant interaction Cr×D (*P*< 0.05). Additionally, each experimental group was compared with the control C by a t-test (^#^ indicates a significant difference vs group C). SEM, pooled standard error of mean (standard deviation for all rats divided by the square root of the number of rats, n = 84). BALP, bone alkaline phosphatase; IFN-γ, interferon-γ; OC, osteocalcin; OCN, osteonectin; M-CSF, macrophage colony-stimulating factor; PGE2, prostaglandin E2; RANK, receptor activator of nuclear factor-κβ; RANKL, receptor activator of nuclear factor-κβ ligand; OPG, osteoprotegerin; RANKL:OPG, RANKL:OPG ratio, Ctn, calcitonin; PTH, parathyroid hormone; Vit D, vitamin D.

**Table 5:** Bone metabolism parameters of rats fed experimental diets*(n = 12).

	BALP	IFN- γ	OC	OCN	M-CSF	PGE2	RANK	RANKL	OPG	RANKL:OPG	Ctn	PTH	Vit D
	ng/g	pg/g	pg/g	ng/g	ng/g	ng/g	ng/g	ng/g	ng/g		pg/g	pg/g	ng/g
Control C	236	965	2057	44.0	78.7	732	344	360	5,191	69.35	352	1995	30.6
t-test/2-way ANOVA:													
M	259^ab#^	861^#^	1972	38.3	77.8	729	268^a#^	361	4,978	72.52c	358	2082^bc#^	24.1^#^
F	217^c^	908^#^	1975	43.5	82.2	684	287^a#^	413^#^	4,845^#^	85.24#a	396^#^	2106^bc#^	33.0
MP	224^c^	772^#^	1974	30.9	68.4^#^	620	179^b#^	386^#^	4,623^#^	83.50#ab	364	2096^bc#^	28.4
FP	218^c^	880^#^	2001	42.1	83.6	697	265^a#^	390^#^	4,827^#^	80.80#b	383^#^	2042^c#^	29.9
MN	237^bc^	883^#^	2172	58.7	79.1	761^#^	217^b#^	416^#^	4,722^#^	88.10#a	499^#^	2191^b#^	30.8
FN	286^a#^	783^#^	2032	47.4	69.6^#^	702	211^b#^	433^#^	4,788^#^	90.43#a	494^#^	2276^a#^	33.7
*SEM*	*4*.*009*	*11*.*879*	*20*.*829*	*2*.*561*	*1*.*069*	*11*.*149*	*7*.*826*	*5*.*409*	*0*,*472*	*1*.*312*	*13*.*601*	*17*.*091*	*0*.*960*
Cr (additional)													
W (without)	238	871^a^	1986	40.2	80.7^a^	713	267	375^b^	4,902	78.88b	371	2062	27.0
P (picolinate)	227	896^a^	2074	51.1	80.6^a^	722	252	414^a^	4,783	82.15ab	447	2149	31.9
N (nano)	255	778^b^	2003	39.2	69.0^b^	661	195	410^a^	4,750	89,20a	429	2186	31.1
*P value*	*0*.*004*	*<0*.*001*	*0*.*264*	*0*.*170*	*<0*.*001*	*0*.*086*	*<0*.*001*	*0*.*005*	*0*.*387*	*0*,*013*	*0*.*056*	*0*.*007*	*0*.*131*
D (Diet type)													
M	233	847	1974	37.5	76.1	678	245	387	4,815	81.37	373	2094	28.5
F	247	849	2068	49.4	77.4	720	231	413	4,808	85.49	458	2170	31.5
*P value*	*0*.*042*	*0*.*933*	*0*.*044*	*0*.*040*	*0*.*515*	*0*.*083*	*0*.*241*	*0*.*013*	*0*.*945*	*0*.*61*	*0*.*002*	*0*.*020*	*0*.*157*
Interaction Cr×D													
*P value*	*<0*.*001*	*0*.*668*	*0*.*287*	*0*.*607*	*0*.*185*	*0*.*096*	*0*.*002*	*0*.*226*	*0*.*155*	*0*.*125*	*0*.*256*	*0*.*022*	*0*.*227*

*The feeding period consisted of an initial 9-wk and an experimental 9-wk period. During the initial period the rats in group C were fed a diet with 8% rapeseed oil and 64% maize starch (diet C), while the remaining groups received a high-fat diet with 8% rapeseed oil, 17% lard, and 52% maize starch (diet F). Dietary treatments used in the experimental period: Group C, control fed diet C; M, fed diet C; F, fed diet F; MP, fed diet Csupplemented with chromium picolinate (CrPic; 0.3 mg/kg body weight (BW)); FP, fed diet F supplemented with CrPic (0.3 mg/kg BW); MN, fed diet C supplemented with chromium nanoparticles (CrNPs; 0.3 mg/kg BW); FN, fed diet F supplemented with CrNPs (0.3 mg/kg BW); W, treatment (n = 24) without Cr supplementation; P, treatment (n = 24) with CrPic supplementation; N, treatment (n = 24) with CrNPs supplementation; M, treatment (n = 36) with standard diet C; F, treatment (n = 36) with high-fat diet F. ^a,b^ Mean values within a column with different superscript letters are significantly different (*P*< 0.05); differences between groups (M, F, MP, FP, MP, MN) are indicated with superscripts only in the case of a statistically significant interaction Cr×D (*P*< 0.05). Additionally, each experimental group was compared with the control C by a t-test (^#^ indicates a significant difference vs group C). SEM, pooled standard error of mean (standard deviation for all rats divided by the square root of the number of rats, n = 84). BALP, bone alkaline phosphatase; IFN-γ, interferon-γ; OC, osteocalcin; OCN, osteonectin; M-CSF, macrophage colony-stimulating factor; PGE2, prostaglandin E2; RANK, receptor activator of nuclear factor-κβ; RANKL, receptor activator of nuclear factor-κβ ligand; OPG, osteoprotegerin; RANKL:OPG, RANKL:OPG ratio, Ctn, calcitonin; PTH, parathyroid hormone; Vit D, vitamin D.

**Table 6:** Gene expression in femur of rats fed experimental diets*(n = 12).

	*SP7*	*RUNX2*	*CTSK*
Control C	1.353	1.455	1.438
t-test/2-way ANOVA:			
M	0.357^ab#^	0.604^#^	0.713^bc#^
F	0.083^c#^	1.053	0.492^c#^
MP	0.148^bc#^	0.955	0.831^bc^
FP	0.366^ab#^	0.812	1.750^a^
MN	0.306^abc#^	0.091^#^	0.441^c#^
FN	0.410^a#^	0.109^#^	1.218^ab^
*SEM*	*0*.*064*	*0*.*087*	*0*.*106*
Cr (additional)			
W (without)	0.220	0.828^a^	0.603
P (picolinate)	0.257	0.883^a^	1.290
N (nano)	0.358	0.100^b^	0.830
*P value*	*0*.*172*	*<0*.*001*	*0*.*015*
D (Diet type)			
M	0.270	0.550	0.662
F	0.286	0.658	1.153
*P value*	*0*.*794*	*0*.*374*	*0*.*012*
Interaction Cr×D			
*P value*	*0*.*005*	*0*.*129*	*0*.*034*

*The feeding period consisted of an initial 9-wk and an experimental 9-wk period. During the initial period the rats in group C were fed a diet with 8% rapeseed oil and 64% maize starch (diet C), while the remaining groups received a high-fat diet with 8% rapeseed oil, 17% lard, and 52% maize starch (diet F). Dietary treatments used in the experimental period: Group C, control fed diet C; M, fed diet C; F, fed diet F; MP, fed diet Csupplemented with chromium picolinate (CrPic; 0.3 mg/kg body weight (BW)); FP, fed diet F supplemented with CrPic (0.3 mg/kg BW); MN, fed diet C supplemented with chromium nanoparticles (CrNPs; 0.3 mg/kg BW); FN, fed diet F supplemented with CrNPs (0.3 mg/kg BW); W, treatment (n = 24) without Cr supplementation; P, treatment (n = 24) with CrPic supplementation; N, treatment (n = 24) with CrNPs supplementation; M, treatment (n = 36) with standard diet C; F, treatment (n = 36) with high-fat diet F. ^a,b^ Mean values within a column with different superscript letters are significantly different (*P*< 0.05); differences between groups (M, F, MP, FP, MP, MN) are indicated with superscripts only in the case of a statistically significant interaction Cr×D (*P*< 0.05). Additionally, each experimental group was compared with the control C by a t-test (^#^ indicates a significant difference vs group C). SEM, pooled standard error of mean (standard deviation for all rats divided by the square root of the number of rats, n = 84). *SP7*,SP7 Transcription Factor, *RUNX2*, *RUNX2*Transcription Factor, *CTSK*, cathepsin K.

### Two-way ANOVA

Two-way ANOVA analysis showed that regardless the Cr addition, the final body weight in rats fed a high-fat diet in both the initial and experimental periods (treatment F) was higher than in rats receiving a high-fat diet only in the initial period and a standard diet in the experimental period (treatment M) [16]. Two-way ANOVA also showed an increase in the levels of OC (*P* = 0.044), OCN (*P* = 0.040),RANKL (*P* = 0.013) and Ctn (*P* = 0.002), in the bones of rats receiving a high-fat diet (F) in both the initial 9-week period and the subsequent 9-week experimental period, in comparison with the rats which receiveda high-fat diet only in the initial periodbut a diet with a standard amount of fat (M) in the experimental period (Table 5).

Two-way ANOVA also showed lower levels of PGE2 (*P* = 0.033), RANKL (*P* = 0.035), RANKL:OPG (*P* = 0.012), PTH (*P* = 0.015), andvit. D (*P* = 0.019) in the plasma (Table 4) and lower levels of IFN-γ and M-CSF (*P*< 0.001, both) with a higher RANKL:OPG ratio (*P* = 0.013) in the bones (Table 5) of rats receiving 0.3 Cr mg/kg diet in the form of CrNPs, compared to rats whose diet was not supplemented with Cr. In addition, irrespective of the form of Cr (CrPic or CrNPs), its addition to the diet ofrats at 0.3 mg/kg of diet resulted in an increased level of RANKL (*P* = 0.005) in the bone tissue compared to rats receiving a diet without the addition of Cr (Table 5). The bones of rats receiving a diet with CrNPs also showed reduced expression of the *RUNX2* gene (*P*< 0.001) compared to rats receiving a diet without added Cr (Table 6).

Two-way ANOVA revealed an interaction for the levels of OC (*P* = 0.039) and RANK (*P* = 0.031) in the plasma of the rats (Table 4),for BALP (*P*< 0.001), RANK (*P* = 0.002) and PTH level (*P* = 0.022) in the bones (Table 5), and for the expression level of the *SP7* (*P* = 0.005) and *CTSK* (*P* = 0.034) genes in the bone tissue (Table 6). The occurrence of these interactions indicates that the main effects had no significant impact on the parameters tested. The interactions observed for the OC level in the plasma (*P* = 0.039) and the PTH level in the bone tissue (*P* = 0.022) were due to the fact that the high-fat diet (F) in combination with the addition of CrNPs to the diet during the experimental period increased the level of OC in the plasma and of PTH in the bones, which was not observed for the use of this diet without Cr supplementation or in combination with the addition of Cr in the form of CrPic. The interactions observed for the RANK level in the plasma (*P* = 0.031) and the level of expression of the *SP7* gene (*P* = 0.005) in the bone tissue were due to the fact that the use of a high-fat diet (F) without added Cr reduced the RANK level in the plasma and the expression of the gene in the bones, which was not observed when this diet was used in combination with the addition of Cr, irrespective of its form (CrNPs or CrPic). The interaction observed for BALP level in the bone tissue (*P*< 0.001) was due to the fact that BALP level was reduced by the use ofa high-fat diet (F) without added Cr but increased by this diet in combination with the addition of Cr in the form of CrNPs, which was not observed in the case of a high-fat F diet with the addition of Cr in the form of CrPic. The interaction observed for the RANK level in the bone tissue (*P* = 0.002) was due to the fact that the use of a high-fat diet (F) in combination with CrPic supplementation during the experimental period increased the RANK level in the bones, which was not observed when this diet was used without Cr or with Crin the form of CrNPs. The interaction observed for expression of the *CTSK* gene in the bone tissue (*P* = 0.034) was due to the fact that the use of a high-fat diet (F) in combination with the addition of CrPic or CrNPs during the experimental period increased the expression of this gene in the bones, which was not observed in the case of the use of this diet without added Cr.

## Discussion

In recent years people have increasingly been abandoning healthy eating habits for a high-fat and low-fibre diet, which provides much more energy than the body can use. The consequence is increasing ratesof obesity, higher than just a few decades ago, when the Western diet was less popular [22]. The results of our earlier research on rats also confirmed that animals receiving a diet with excessive content of saturated fats attained a significantly higher final body weight and accumulated significantly more adipose tissue than animals with a normal diet [16]. The literature indicates that obesity, including obesity induced by chronic intake of a high-fat diet, may be accompanied by the development of numerous disorders, such as metabolic syndrome, insulin resistance, diabetes, cardiovascular disease, and even cancers [23–25]. There are also reports that this type of diet affects the structure and health of the skeletal system, although opinions on the direction of this effect are clearly divided [25,26]. On the one hand, it has been suggested that excess adipose tissue deposited during intake of a high-fat diet, by increasing mechanical load, may stimulate bone-formation processes, leading to the achievement of peak bone mass and reducing the risk of osteoporosis [2,3]. It is also supposed that the beneficial stimulation of bone-formation processes observed during the use of a high-fat diet (HFD) may be due to the fact that white adipose tissue (WAT), which is then accumulated in excess, performs endocrine functions in the body and is able to secrete significant amounts of adipokines, especially leptin and adiponectin, which exert a direct anabolic effect onosteoblasts [3]. Both of these hormones stimulate the proliferation of bone marrow stromal cells, osteoblast differentiation, and mineralization processes through increased expression of the bone-specific isoform of alkaline phosphatase (ALP), osteocalcin (OC) andtype I collagen, which indicates that they are able to effectively stimulate bone-formation processes [27–29]. Leptin also inhibits osteoclastogenesis, thereby preventing bone resorption [30]. The literature indicates that this hormone increases the expression of osteoprotegerin (OPG), which by binding RANK ligand (RANKL, receptor activator of nuclear factor-κβligand) prevents it from binding to RANK (receptor activator of nuclear factor-κβ), located on the surface of osteoclasts, thereby preventing their activation and the initiation of osteolytic processes [30]. Studies by Burguer et al. [31] and Reid et al. [32] indicate that the presence of leptin secreted by WAT may also inhibit bone resorption by directly reducing the expression of RANKL.

On the other hand, in recent years there have been increasing reports indicating that excessive consumption of an obesogenic Western diet adversely affects bone turnover, which negatively affects the functioning and structure ofbone tissue. In effect,the skeleton becomes more susceptible to micro damage and fractures [5,26,33,34]. Ionova-Martin et al. [35] demonstrated that although obesity caused by a high-fat diet may be accompanied by increased bone tissue mass due to increased bone size and mineral content, its quality deteriorates as well (poorer mechanical properties). Cao et al. [33], in a study on mice, showed that a high-fat diet induces osteoclast maturation and activity and increases bone resorption, as indicated by an increase in RANKL expression, the RANKL:OPG ratio, and the level of the bone-specific isoform ofalkaline phosphatase (TRAP). The results of the present study also showed that feeding rats a high-fat/low-fibre diet negatively affects the RANK/RANKL/OPG pathway. In the bones of these rats there was a decrease in the OPG and RANK levels accompanied by an increase in expression of RANKL. RANKL is a regulatory molecule produced by osteoblasts, which by binding to RANK (receptor activator of nuclear factor-κβ) on the surface of osteoclast precursors triggers a signalling cascade within the maturing osteoclast, which leads to the formation of a fully active bone-resorbing cell. OPG, also produced by osteoblasts, is a protein functioning as an inhibitor of osteoclasto genesis. Its binding to RANKL prevents the RANK/RANKL interaction, there by stopping the entire osteoclast maturation pathwayin its initial stages [36]. The results of the present study therefore indicate that the use of a HFD led to a marked increase inosteoclasto genesis, negatively affecting total bone turnover. This supposition is also confirmed by the RANKL:OPG ratio, which points to an imbalance in the levels of these molecules, with a significant predominance of RANKL. Interestingly, both RANK and RANKL were decreased in the rats with no changes in the OPG level, which may be due to their local accumulation in the bones. The results of our study also showed a decrease in IFN-γ levels in both the bloodandbonesof rats feda high-fat diet. The literature indicates that thiscytokineshows a tendency to inhibit bone resorption processes [2]. Its reduced level accompanied by an increased RANK level therefore seems to additionally confirm that an obesogenic diet induces excessive bone resorption.

Our previous research showed reduced plasma levels of Ca and P induced by the use ofa high-fat diet [13]. The present study additionally showed that although there were no significant changes in the level of vitamin D_3_ in the bones of obese rats, there was a marked decrease in its level in the blood. This seems to be in agreement with reports by Hou et al. [3] and Marques Loureiro et al. [5], who observed that chronic intake of an unbalanced high-fat diet can contribute to disorders in bone modelling and remodelling by increasing the risk of deficiencies of important nutrients involved in bone metabolism, such as calcium, phosphorus, and vitamin D_3_. Ca and P are essential tohydroxyapatite synthesis andbone mineralization, ensuring adequate bone strength [37]. Sufficient amounts of calcium and phosphates fornormal mineralizationcan be ensured by the active form ofvitamin D_3_,regarded as a factor activating osteoclast maturation processes by increasing the expression of RANKL and reducing OPG levels [2]. In addition, activevitamin D_3_ has been shown to have a direct impact on osteoblasts, increasing production of osteocalcin and osteopontin as well as proteins involved in bone mineralization [38]. In the present study, the decrease in thevitamin D_3_ level in the blood without changes in its level in thebones, as well as the increased RANKL level in the bones, may additionally suggest that it was locally taken up and used by intensively resorbedbone tissue.

Marques Loureiro et al. [5] also note that obesity induced by a high-fat diet is often accompanied by metabolic disorders leading to hormonal imbalance. This may be manifested as a reduced level of parathormone (PTH), which regulates the calcium–phosphate balance in the body [6]. The results of our study, however, showed an increased PTH level in thebonesand a reduced level in the plasma. PTH is produced by the parathyroid glands in response to a decline in the Ca level in the blood. It stimulates the release of calcium from the bones to the blood in the bone tissue resorption process. This is in agreement with the results of our earlier research, in which the use ofa high-fat dietin ratsreduced the Ca level in the blood [13]. The activity of PTH includes stimulation of osteoblaststo increase RANKL production as well as inhibition of the secretion of OPG, enabling the differentiation, maturation and activation of osteoclasts. This results in the release of Ca and P into the blood due to the dissolution and degradation ofhydroxyapatite and other organic material in the bones [6]. In addition, PTH increases Ca absorption from the gastrointestinal tract and increases renal Ca reabsorption. When the level of calcium in the body reaches an adequate level, the production and release of PTH stop. A long-termincrease in the PTH levelincreases the rate of bone remodelling and may cause bone loss [38]. Interestingly, the results of our study showed a simultaneous increase in the calciton in level in the bones of obese rats. This is surprising, because calcitonin is antagonistic to PTH, so when the level of one increases, the level of the other would be expected to decrease. This is because Ctn is produced by the thyroid when the Ca level in the blood becomes too high. This hormoneremoves excess calcium from the blood by intensifying bone formation processes, thus increasing its deposition in bone tissue, and also reduces intestinal absorption and renal reabsorption of Ca [39]. However, it has been demonstrated that at normalCa levels in the body, PTH and Ctn can co-exist and complement one another [10]. Nevertheless, given that in our earlier research the plasma Ca and P levels were reduced in HFD-fed rats (Stępniowska et al., 2022), the increased Ctn level in the bones of obese rats in the present study is surprising and difficult to explain.

Normal bone modelling and remodelling are also significantly influenced by adequate *CTSK* mRNA expression, which translates to synthesis of the functional protein cathepsin K. This protein belongs to thepapain family of cysteineproteases with the ability to digest type I collagen, and itsexpressionis characteristic of osteoclasts [40]. Halade et al. [34] demonstrated that a high-fat diet contributes to an unfavourable increase in the expression of CTSK in mice, while the results of the present study showed a decrease in *CTSK* mRNA expression in the bones of obese rats. In the light of our previously cited results, as well as the results of research by Halade et al. [34], the decrease in *CTSK* mRNA expression observed in the present study may be due to the use and rapid depletion of the transcript for synthesis of this functional protein involved in intensive bone degradation.The results of our study additionally showed reduced *SP7* and *RUNX2* mRNA expression in the bones of rats receiving a high-fat diet. This is in agreement with the findings of Halade et al. [34] and Ross et al. [41], whose research in mice also confirmed a negative effect of a HFD onexpression of *RUNX2*. *Runx2* and *SP7/Osterix* play an important role in bone formation as essential transcription factors for the differentiation of mesenchymal cells to chondrocytesin cartilage and osteoblastsin bone. It is generally believed that the transformation of progenitor mesenchymal cells into preosteoblasts requires *RUNX2*, and further differentiation into a mature osteoblast is regulated by the coordinated action of*RUNX2* and *SP7* [41]. Ross et al. [41] suggest that the reduced expression of *RUNX2* noted in their study, accompanied by reduced expression of Ocnin HFD-fed mice, is indicative of inhibition of osteoblast activity, which may lead to deterioration of bone quality and increased fragility. Similarly, in the present study, in addition to reduced *RUNX2* and *SP7* mRNA expression in the bones of obese rats, Ocn and BALP levels were reduced as well. Ocnis secreted by osteoblasts, and its expression is directly linked to their differentiation and mineralization. Therefore the decreased Ocn level suggests that osteoblasts may not be fully differentiated ormay be quiescent [41]. BALP, anchored in the membrane of osteoblasts and matrix vesicles, plays an important role in bone mineralization by promoting the growth of hydroxyapatite crystals, and its concentration in the serum increases during rapid growth and bone remodelling [42]. In light of the above, it can be concluded that the use of a HF diet not only intensified bone resorption processes but also impaired bone synthesis, which can cause a bone turnover imbalance leading to excessive loss of bone mass.

The literature indicates that metabolic disorders associated with obesity developing due to chronic intake of a diet with high content of saturated fats can be at least partially mitigated by a return to a healthy, balanced diet [4,16,43]. Although our previous research showed that switching from obesogenic dietary habits to a healthy, balanced diet resulted in a beneficial reduction in adipose tissue and weight gain, and thus to a significantly lower final body weight in rats [16], the present study showed that this treatment effectively reduced the RANK level in the blood and BALP activity in the bones, while increasing *SP7* mRNA expression in the bones. Although the increase in *SP7* mRNA expression in the bones of rats may indicate stimulation of bone formation processes, in light of the other results it seems likely that the switch to healthy eating habits was insufficient to regulate the bone turnover disturbances induced by an obesogenic diet.

In our experiment we also tested the effect of a change in eating habits from an obesogenic diet to a healthy, balanced diet which is additionally modified by supplementation with the recommended pharmacologically active dose of Cr(III), in the form of picolinate or of nanoparticles, which are of increasing interest among scientists. The literature indicates that Cr(III) reduces the level of osteocalcin, which in excessive amounts can contribute to osteoporosis [9]. In addition, this microelement acts on the bones by modulating the activity of alkaline phosphatase (ALP) and tartrate-resistant acidphosphatase (TRAP) [10]. Supplementation with Cr(III) has also been shown to prevent osteoporosis in postmenopausal women [8]. Moreover, according to the literature, the addition of Cr to the dietincreases Ca retention, suggesting improvement in bone mineralization [8]. However, there are also reports that chromium can adversely affect bone functioning and structure [10]. The results of our previous research in chickens and rats [12,13] showed that Cr added to the diet may accumulate in the bones and at the same time reduce their content of phosphorus. Moreover, Saeed et al. [14] report that Cr can displace other elements from the bones, including Ca, by becoming incorporated in their place, which negatively affects bone properties. Furthermore, by increasing insulin activity in the bones, Cr can inhibit resorption processes by blocking the activity of parathormone on osteoblasts [8]. The results of our study showed that the addition of CrPic only enhanced the reduction in BALP activity in the bones induced by switching from a high-fat diet to a standard diet. This enzyme is involved in bone formation, and its activity is particularly high during intensive skeletal growth and remodelling [44]. Roczniak et al. [10] noted that a decline in the activity of alkaline phosphatase often accompanies significant chromium accumulation in the skeleton, which adversely affects the rate of bone formation. Thus the progressive decline observed in the BALP level can be interpreted as an indicator of inhibition of the bone formation process by the addition of CrPic. However, the absence of accompanying changes in the levels of other markers of bone formation relative to the animals for which only the diet was changed suggests that CrPic had no significant impact on these processes, either positive or negative, and its reduction may be due to other factors, such as temporary local use of BALP to repair microdamage. In addition, the results of our study showed that the switch from obesogenic to healthy eating habits in combination with the addition of a recommended pharmacologically active dose of Cr in the form of CrNPs resulted in an increase in IFN-γ levels in the blood of rats, while its levels in the bones remained unchanged. This protein is synthesized mainly by T lymphocytes, but also by NK cells in response to a foreign antigen. The primary role of IFN-γ is to activate macrophages to produce pro-inflammatory cytokinesand stimulate the immune response by activating B and T cells. Their effect onbone tissue involves stimulating osteolysis by increasing RANKL expression. However, the absence of changes in the level of either IFN-γ in the bones or RANKL in the bones or plasma may suggest that the addition of CrNPs did not activate osteoclastogenesis processes. This also seems to confirm the reduced RANK expression observed in the bones during administration of chromium to rats, in the form of CrNPs or CrPic. This is in contrast to the results of an *in vitro* study conducted by Chen et al. [45], in which CrNPs impaired osteogenic differentiation of MSCs and reduced osteogenic expression of the *OPN*, *COX2* and *RUNX2* genes. The differences between the results obtained by Chen et al. [45] and our own may be due to the amount of nanoparticles used, their size, and especially the *in vivo* vs *in vitro* experimental models.

The present study, however, showed that the addition of Cr(III) (CrNPs or CrPic) affects bone turnover not only in the case of a switch to healthy dietary habits, but also when this change was not introduced, and the rats continued to be fed an obesogenic diet. In effect, the addition of CrPic to a high-fat diet reduced only the IFN-γ level in the blood of rats. Thus it seems likely that Cr supplementation in the form of picolinatein the dose applied, with or without a change in diet, did not mitigate the unfavourable changes in the level of biochemical markers caused by an obesogenic diet, but also did not exacerbate them. On the other hand, the use of CrNPs in rats fed a high-fat diet increased the OC and RANK levels in the blood while increasing PTH and BALP levels and reducing RANK levels in the bones. The increase in thelevel of BALP in the bones and OC in the plasma, together with a decrease in the RANK level in the bones, suggests an intensification of bone formation processes, while the increase in PTH in the bones may suggest the initiation of osteoclastogenesis aimed at balancing osteogenesis and regulating bone turnover at an appropriate level. In light of the results cited above, CrNPs can be presumed to have a beneficial effect on bone metabolism only when rats continue to be fed an obesogenic diet. Moreover, the addition of chromium to a high-fat obesogenic diet, irrespective of its form, increased *SP7* and *CTSK* genes expression in the bones, with CrPi chaving a stronger effect on *CTSK*, and CrNPs on *SP7*. Thus bone metabolic processes were stimulated at the molecular level in obese rats by both forms of Cr, with CrPic apparently directing them towards osteoclastogenesis (bone resorption), and CrNPs towards osteoblastogenesis (bone formation). The results conflict with those obtained by Chen et al. [45], who found that CrNPs adversely affect bones by reducing osteogenic differentiation and the expression of important genes involved in bone formation processes. As few studies have been carried out to establish the mechanism of action of Cr(III), especially CrNPs, on bone turnover, our results are difficult to interpret.

To sum up, the results of the study showed that chronic intake ofa high-fat obesogenic diet negatively affects bone turnover by impairing processes of both synthesis and degradation of bones. The switch to a healthy diet proved insufficient to regulate bone metabolism disorders induced by an obesogenic diet, even when it was supplemented with chromium, irrespective of its form. Supplementation with CrPic with no change in diet stimulated bone metabolism only at the molecular level, towards increased osteoclastogenesis (bone resorption). In contrast, CrNPs added to the high-fat diet effectively regulated bone turnover by increasing both osteoblastogenesis and osteoclastogenesis, with these changes directed more towards bone formation. The results of the study suggest that unfavourable changes in bone metabolism induced by chronic intake of a high-fat diet can be mitigated by supplementation with CrNPs, whereas a change in eating habits fails to achieve a similar effect.

## References

[1] Lac G, Cavalie H, Ebal E, Michaux O. Effects of a high fat diet on bone of growing rats. Correlations between visceral fat, adiponectin and bone mass density. Lipids Health Dis. 2008;7:16. doi: 10.1186/1476-511X-7-16 18442361 PMC2386795

[2] Stanisławowski M, Kmieć K. The participation of RANK, RANKL and OPG in tumor osteolysis. PostepyHig Med Dosw. 2009; 63: 234–241. (in Polish) 19502684

[3] Hou J, He C, He W, Yang M, Luo X, Li C. Obesity and Bone Health: A Complex Link. Front Cell Dev Biol. 2020;8: 600181. doi: 10.3389/fcell.2020.600181 33409277 PMC7779553

[4] Qiao J, Wu Y, Ren Y. The impact of a high fat diet on bones: potential mechanisms. Food Funct. 2021;12(3): 963–975. doi: 10.1039/d0fo02664f 33443523

[5] Marques Loureiro L, Lessa S, Mendes R, Pereira S, Saboya CJ, Ramalho A. Does the Metabolically Healthy Obese Phenotype Protect Adults with Class III Obesity from Biochemical Alterations Related to Bone Metabolism? Nutrients. 2019;11(9): 2125. doi: 10.3390/nu11092125 31489911 PMC6771134

[6] Khan M, Jose A, Sharma S. Physiology, Parathyroid Hormone. 2022. In: StatPearls [Internet]. Treasure Island (FL): StatPearls Publishing; 2023. 29763115

[7] Dworzański W, Cholewińska E, Fotschki B, Juśkiewicz J, Listos P, Ognik K. Assessment of DNA Methylation and Oxidative Changes in the Heart and Brain of Rats Receiving a High-Fat Diet Supplemented with Various Forms of Chromium. Animals (Basel). 2020;10(9): 1470. doi: 10.3390/ani10091470 32825649 PMC7552180

[8] McCarty MF. Anabolic effects of insulin on bone suggest a role for chromium picolinate in preservation of bone density. Med Hypotheses. 1995;45(3): 241–246. doi: 10.1016/0306-9877(95)90112-4 8569546

[9] Allen MJ, Myer BJ, Millett PJ, Rushton N. The effects of particulate cobalt, chromium and cobalt-chromium alloy on human osteoblast-like cells in vitro. J Bone Joint Surg Br. 1997;79(3): 475–482. doi: 10.1302/0301-620x.79b3.7415 9180332

[10] Roczniak W, Brodziak-Dopierała B, Cipora E, Jakóbik-Kolon A, Konieczny M, Babuśka-Roczniak M. Analysis of the Content of Chromium in Certain Parts of the Human Knee Joint. Int J Environ Res Public Health. 2018;15(5): 1013. doi: 10.3390/ijerph15051013 29772846 PMC5982052

[11] Haq Z, Jain RK, Khan N, Dar MY, Ali S, Gupta M, et al. Recent advances in role of chromium and its antioxidant combinations in poultry nutrition: A review. Vet World. 2016;9(12): 1392–1399. doi: 10.14202/vetworld.2016.1392-1399 28096611 PMC5234053

[12] Stępniowska A, Tutaj K, Drażbo A, Kozłowski K, Ognik K, Jankowski J. Estimated intestinal absorption of phosphorus and its deposition in chosen tissues, bones and feathers of chickens receiving chromium picolinate or chromium nanoparticles in diet. PLoS One. 2020;15(11): e0242820. doi: 10.1371/journal.pone.0242820 33237949 PMC7688154

[13] Stępniowska A, Juśkiewicz J, Tutaj K, Fotschki J, Fotschki B, Ognik K. Effect of Chromium Picolinate and Chromium Nanoparticles Added to Low- or High-Fat Diets on Chromium Biodistribution and the Blood Level of Selected Minerals in Rats. Polish J Food Nutr Sci. 2022;72(3): 229–238.

[14] Saeed AA, Sandhu MA, Khilji MS, Yousaf MS, Rehman HU, Tanvir ZI, et al. Effects of dietary chromium supplementation on muscle and bone mineral interaction in broiler chicken. J Trace Elem Med Biol. 2017;42: 25–29. doi: 10.1016/j.jtemb.2017.03.007 28595787

[15] Stępniowska A, Tutaj K, Juśkiewicz J, Ognik K. Effect of a high-fat diet and chromium on hormones level and Cr retention in rats. J Endocrinol Invest. 2022;45(3): 527–535. doi: 10.1007/s40618-021-01677-3 34550535 PMC8850218

[16] Fotschki B, Ognik K, Fotschki J, Napiórkowska D, Cholewińska E, Krauze M, et al. Chromium Nanoparticles Together with a Switch Away from High-Fat/Low-Fiber Dietary Habits Enhances the Pro-Healthy Regulation of Liver Lipid Metabolism and Inflammation in Obese Rats. Int J Mol Sci. 2023;24(3): 2940. 36769261 10.3390/ijms24032940 PMC9918060

[17] Fotschki B, Ognik K, Cholewińska E, Grzelak-Błaszczyk K, Myszczyński K, Krauze M, Juśkiewicz J. Effect of Chromium Nanoparticles and Switching from a High-Fat to a Low-Fat Diet on the Cecal Microenvironment in Obese Rats. Nutrients. 2023;15(14): 3118. doi: 10.3390/nu15143118 37513536 PMC10384463

[18] Directive 2010/63/EU of the European Parliament and of the Council of 22 September 2010 on the protection of animals used for scientific purposes. OJEU. 2010;L276,20.10.2010: 33–79.

[19] Reeves PG. Components of the AIN-93 diets as improvements in the AIN-76 A diet. J Nutr. 1997;127(5 Suppl): 838S-841S. 10.1093/jn/127.5.838S 9164249

[20] EFSA NDA Panel (EFSA Panel on Dietetic Products, Nutrition and Allergies). Scientific Opinion on Dietary Reference Values for chromium. EFSA J. 2014;12: 3845. 10.2903/j.efsa.2014.3845 PMC13136993 42087961

[21] Nance R, Agarwal P, Sandey M, Starenki D, Koehler J, Sajib AM, et al. A method for isolating RNA from canine bone. Biotechniques. 2020;68(6): 311–317. doi: 10.2144/btn-2019-0153 32301333

[22] Wali JA, Jarzebska N, Raubenheimer D, Simpson SJ, Rodionov RN, O’Sullivan JF. Cardio-Metabolic Effects of High-Fat Diets and Their Underlying Mechanisms-A Narrative Review. Nutrients. 2020;12(5): 1505. doi: 10.3390/nu12051505 32455838 PMC7284903

[23] Albadah MS, Dekhil H, Shaik SA, Alsaif MA, Shogair M, Nawaz S, et al. Effect of weight loss on serum osteocalcin and its association with serum adipokines. Int J Endocrinol. 2015;2015:508532. doi: 10.1155/2015/508532 25784935 PMC4345075

[24] Alsaggar M, Mills M, Liu D. Interferon beta overexpression attenuates adipose tissue inflammation and high-fat diet-induced obesity and maintains glucose homeostasis. Gene Ther. 2017;24(1): 60–66. doi: 10.1038/gt.2016.76 27858942 PMC5757862

[25] Zhu R, Wang Z, Xu Y, Wan H, Zhang X, Song M, et al. High-Fat Diet Increases Bone Loss by Inducing Ferroptosis in Osteoblasts. Stem Cells Int. 2022;2022: 9359429. doi: 10.1155/2022/9359429 36277036 PMC9586793

[26] Song W, Sheng Q, Bai Y, Li L, Ning X, Liu Y, et al. Obesity, but not high-fat diet, is associated with bone loss that is reversed via CD4+CD25+Foxp3+ Tregs-mediated gut microbiome of non-obese mice. NPJ Sci Food. 2023;7(1): 14. doi: 10.1038/s41538-023-00190-6 37055440 PMC10102288

[27] Thomas T, Gori F, Khosla S, Jensen MD, Burguera B, Riggs BL. Leptin acts on human marrow stromal cells to enhance differentiation to osteoblasts and to inhibit differentiation to adipocytes. Endocrinology. 1999;140(4): 1630–1638. doi: 10.1210/endo.140.4.6637 10098497

[28] Luo XH, Guo LJ, Yuan LQ, Xie H, Liao EY. Adiponectin stimulates human osteoblasts proliferation and differentiation via the MAPK signaling pathway. Chin J Osteoporos. 2005;309: 99–109. doi: 10.1016/j.yexcr.2005.05.021 15963981

[29] Astudillo P, Ríos S, Pastenes L, Pino AM, Rodríguez JP. Increased adipogenesis of osteoporotic human-mesenchymal stem cells (MSCs) characterizes by impaired leptin action. J Cell Biochem. 2008;103(4): 1054–1065. doi: 10.1002/jcb.21516 17973271

[30] Holloway WR, Collier FM, Aitken CJ, Myers DE, Hodge JM, Malakellis M, et al. Leptin inhibits osteoclast generation. J Bone Miner Res. 2002;17(2): 200–209. doi: 10.1359/jbmr.2002.17.2.200 11811550

[31] Burguera B, Hofbauer LC, Thomas T, Gori F, Evans GL, Khosla S, et al. Leptin reduces ovariectomy-induced bone loss in rats. Endocrinology. 2001;142: 3546–3553. doi: 10.1210/endo.142.8.8346 11459801

[32] Reid IR, Baldock PA, Cornish J. Effects of Leptin on the Skeleton. Endocr Rev. 2018;39(6): 938–959. doi: 10.1210/er.2017-00226 30184053

[33] Cao JJ, Gregoire BR, Gao H. High-fat diet decreases cancellous bone mass but has no effect on cortical bone mass in the tibia in mice. Bone. 2009;44(6): 1097–1104. doi: 10.1016/j.bone.2009.02.017 19264159

[34] Halade GV, El Jamali A, Williams PJ, Fajardo RJ, Fernandes G. Obesity-mediated inflammatory microenvironment stimulates osteoclastogenesis and bone loss in mice. Exp Gerontol. 2011;46(1): 43–52. doi: 10.1016/j.exger.2010.09.014 20923699 PMC2998554

[35] Ionova-Martin SS, Do SH, Barth HD, Szadkowska M, Porter AE, Ager JW III, et al. Reduced size-independent mechanical properties of cortical bone in high-fat diet-induced obesity. Bone. 2010;46(1): 217–225. doi: 10.1016/j.bone.2009.10.015 19853069 PMC4501030

[36] Mikoś H, Mikoś M, Mikoś M, Obara-Moszyńska M, Niedziela M. The role of the OPG/RANKL/RANK system in obesity in children and adolescents. NowinyLekarskie, 2010; 79(5): 403–409. (In Polish)

[37] Zastulka A, Clichici S, Tomoaia-Cotisel M, Mocanu A, Roman C, Olteanu CD, et al. Recent Trends in Hydroxyapatite Supplementation for Osteoregenerative Purposes. Materials (Basel). 2023;16(3):1303. doi: 10.3390/ma16031303 36770309 PMC9919169

[38] Bellido T. Osteocyte-driven bone remodeling. Calcif Tissue Int. 2014;94(1): 25–34. doi: 10.1007/s00223-013-9774-y 24002178 PMC3947228

[39] Christenson RH. Biochemical markers of bone metabolism: an overview. Clin Biochem. 1997;30(8): 573–593. doi: 10.1016/s0009-9120(97)00113-6 9455610

[40] Costa A, Cusano N, Silva B, Cremers S, Bilezikian JP. Cathepsin K: its skeletal actions and role as a therapeutic target in osteoporosis. Nat Rev Rheumatol.2011;7: 447–456. doi: 10.1038/nrrheum.2011.77 21670768

[41] Ross DS, Yeh TH, King S, Mathers J, Rybchyn MS, Neist E, et al. Distinct Effects of a High Fat Diet on Bone in Skeletally Mature and Developing Male C57BL/6J Mice. Nutrients. 2021;13(5): 1666. doi: 10.3390/nu13051666 34068953 PMC8157111

[42] Nizet A, Cavalier E, Stenvinkel P, Haarhaus M, Magnusson P. Bone alkaline phosphatase: An important biomarker in chronic kidney disease—mineral and bone disorder. Clin Chim Acta. 2020;501: 198–206. doi: 10.1016/j.cca.2019.11.012 31734146

[43] Braga SP, Delanogare E, Machado AE, Prediger RD, Moreira ELG. Switching from high-fat feeding (HFD) to regular diet improves metabolic and behavioral impairments in middle-aged female mice. Behav Brain Res. 2021;398: 112969. doi: 10.1016/j.bbr.2020.112969 33075395

[44] Tariq S, Tariq S, Lone KP, Khaliq S. Alkaline phosphatase is a predictor of Bone Mineral Density in postmenopausal females. Pak J Med Sci. 2019;35(3): 749–753. doi: 10.12669/pjms.35.3.188 31258588 PMC6572960

[45] Chen J, Ding J, Wu Y, Zhang S, Zheng N, Yang J, et al. Chromium Oxide Nanoparticle Impaired Osteogenesis and Cellular Response to Mechanical Stimulus. Int J Nanomedicine. 2021;16: 6157–6170. doi: 10.2147/IJN.S317430 34511912 PMC8423495

